# The Parametric Study and Fine-Tuning of Bow-Tie Slot Antenna with Loaded Stub

**DOI:** 10.1371/journal.pone.0169033

**Published:** 2017-01-23

**Authors:** M. M. Shafiei, Mahmoud Moghavvemi, Wan Nor Liza Wan Mahadi

**Affiliations:** 1 Department of Electrical Engineering, Faculty of Engineering, University of Malaya (UM), Kuala Lumpur, Malaysia; 2 Center of Research in Applied Electronics (CRAE), Faculty of Engineering, University of Malaya (UM), Kuala Lumpur, Malaysia; 3 University of Science and Culture, Tehran, Iran; West Virginia University, UNITED STATES

## Abstract

A printed Bow-Tie slot antenna with loaded stub is proposed and the effects of changing the dimensions of the slot area, the stub and load sizes are considered in this paper. These parameters have a considerable effect on the antenna characteristics as well as its performance. An in-depth parametric study of these dimensions is presented. This paper proposes the necessary conditions for initial approximation of dimensions needed to design this antenna. In order to achieve the desired performance of the antenna fine tuning of all sizes of these parameters is required. The parametric studies used in this paper provide proper trends for initiation and tuning the design. A prototype of the antenna for 1.7*GHz* to 2.6*GHz* band is fabricated. Measurements conducted verify that the designed antenna has wideband characteristics with 50% bandwidth around the center frequency of 2.1*GHz*. Conducted measurements for reflection coefficient (*S*_*11*_) and radiation pattern also validate our simulation results.

## Introduction

As can be understood from many published works, various multiple communication techniques play a major role to improve the reliability and performance of the contemporary communication system. One of the techniques to support the increasing demand for the long range data communication is using the long-haul fiber-optic communication systems [1] and wavelength-division multiplexed systems [2]. Another invented multiple communication techniques as an interesting major of new researches for next generation communication systems are multiple-antenna systems and multiuser communications [3].

Recent and growing developments in wireless communication systems require wideband, low profile, small size, lightweight and low-cost antennas [4–9]. Low profile patch antennas such as microstrip and printed slot antenna meet these requirements. Nevertheless, a well-known drawback of the patch antenna is its intrinsic narrow bandwidth. Although different shapes of printed monopole antennas such as meander shaped [10], modified square-shaped [11], inverted double L-shaped [12], modified Inverted-L shaped [13], twin stepped patch [14], double-T shaped [15] and arc-shaped [16] are employed, they cannot provide a large enough bandwidth to cover wideband applications.

Rapid developments of wireless communication technologies generally require two-way data communication in a fast, high-efficiency and reliable way, which is reflected in the antenna subsystem [17]. A dual band H-slot microstrip patch antenna is presented to cover two frequency bands of 3.6–3.7 *GHz* and 5.7–5.8 *GHz* [17]. Another published work presented a low-profile, single layered rectangular microstrip antenna for 2.45 *GHz* and 4.96 *GHz* bands [18]. Although some of designs may provide dual or multi-band operations, the bandwidth around each resonant frequency is not large enough. Therefore, there are a variety of techniques to be applied and increase the bandwidth of the antenna. A slotted antenna is one of the effective candidates for exhibiting wideband characteristics by the printed antenna [19–23]. Desired bandwidth can be achieved by tuning the input impedance of the antenna by means of varying slot dimensions or geometry [24], using different patch shapes or using notched ground plane. The latter creates a capacitive load that compensates the natural inductive load of the monopole and makes the input impedance nearly-resistive [19]. A novel design presented a probe feed miniaturized dual-band microstrip antenna fabricated on a single layer patch. It used an asymmetric J Slot to achieve its desired characteristics [25].

One of the wideband antenna geometries is the Bow-Tie antenna that can be fed by coaxial line [26, 27], coplanar waveguide [28, 29] and stripline [30]. It has the advantages of low profile antennas with wideband characteristics and high radiation efficiency. The Bow-Tie antenna has been used in many applications such as WiFi [31] ground penetrating radars (GPR) [32–34] and pulse antennas [26, 27, 35]. Literature review for the Bow-Tie slot antennas demonstrates the wideband characteristics of this structure [29, 36–40].

The simple structure as well as the performance of the antenna are important issues in the design. The simple-structure wideband antenna has been realized by both microstrip line feeding structure and coplanar waveguide (*CPW*) feeding structure [19]. The antenna for this purpose should provide an easy means of fabrication and installation to facilitate parallel and series connections with circuit elements (for matching and gain enhancement) and easy integration with Monolithic Microwave Integrated Circuits (*MMIC*s) [41, 42]. CPW-fed printed slot antenna provides easy means of integration with *MMIC*s [43] in addition to its advantages for demonstrating the wider bandwidth as well as less radiation loss and less dispersion [19, 29].

There are some publications that they mainly has studies the characteristics of dual- or multi-band microstrip antennas and experimentally described how satisfying are their performance [17]. In this paper, a printed Bow-Tie slot antenna fed by a coplanar waveguide and directly matched to the 50*Ω* SMA connector for wide bandwidth applications has been presented and discussed. The antenna is carefully analyzed to determine the effects of all dimensions on the reflection coefficients in the working frequency band. The analysis and assessment of the antenna is performed by the CST Microwave StudioTM. This computer simulation software package employs the Finite Integration Technique for electromagnetic computation. Simulation results provide a good initial approximation of antenna dimensions and suggest proper tuning trends to achieve desired performance. The radiation patterns of the antenna in E and H planes are presented. The antenna prototype is built by generic printed circuit board (*PCB*) technologies, and measurement results prove the validity of our simulations.

## Antenna Design

**Fig 1:**
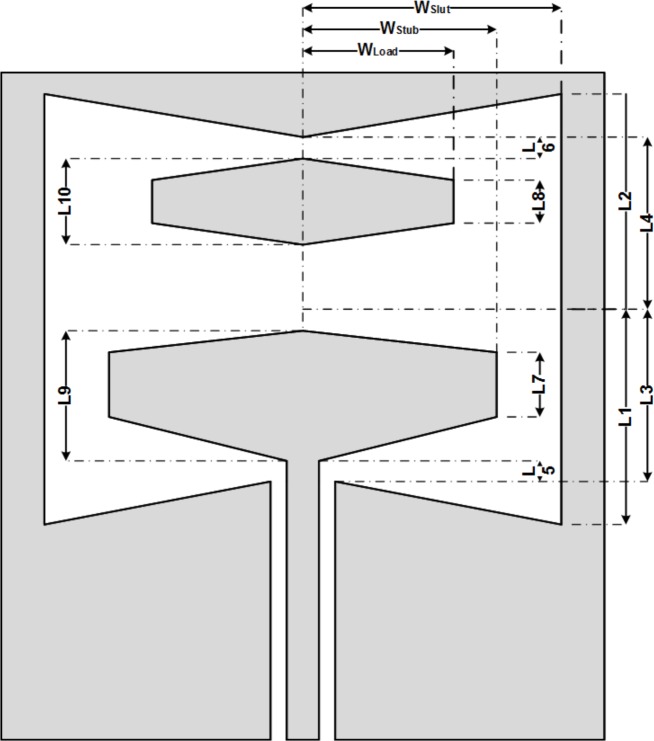
Geometry of the Proposed Bow-Tie Antenna.

Coplanar waveguide (*CPW*) feeding with loaded stub used for the antenna and some modifications have been applied to the geometry to comply with our desired performance and characteristics. The proposed geometry is shown in Fig 1. The parametric design of the antenna provides more degrees of freedom for tuning purposes during simulation and parametric study during the analysis. The width and length of the Bow-Tie slot next to the tuning of the stub and load define the effective parameters. Theses sizes must be taken into account for a full analysis of the antenna.

The dimensions of the antenna are presented by variables such as, width of the slot (*W*_*Slot*_), width of the stub (*W*_*Stub*_), width of the load (*W*_*Load*_), length of the slot (*L*_*Slot*_), aperture of the slot (*A*_*Slot*_), length of the stub (*L*_*Stub*_), aperture of the stub (*A*_*Stub*_), length of the load (*L*_*Load*_), aperture of the load (*A*_*Load*_), length of the first gap (*L*_*G1*_) and length of the second gap (*L*_*G2*_). These variables determine the size and geometry of the proposed antenna. The sizes used for our fabrication are: (All sizes are in millimeter)

*W*_*Slot*_ = 44 *L*_*Slot*_ = 32 *L*_*Stub*_ = 4 *L*_*Load*_ = 3
*W*_*Stub*_ = 32 *A*_*Slot*_ = 21 *A*_*Stub*_ = 8 *A*_*Load*_ = 7
*W*_*Load*_ = 20 *L*_*G1*_ & *L*_*G2*_ = 0.5
*W*_*substrate*_ = 55 *L*_*substrate*_ = 54

In this design *W*_*Slot*_ is the Bow-Tie slot width. The changes of *W*_*Slot*_ presents changes in the width of the slot area. Also *L*_*Slot*_ is the Bow-Tie length and the changing of *L*_*Slot*_ presents changes in the length of the slot area. The *A*_*Slot*_ determines the indent size of the Bow-Tie and shows the inner aperture of the slot.

The stub and the load are located inside the patch area. The *W*_*Stub*_ presents the width of the stub embedded inside the Bow-Tie slot area and width of the load object is presented by *W*_*Load*_. The other parameters like *L*_*Stub*_, *A*_*Stub*_, *L*_*Load*_ and *A*_*Load*_ present the length and aperture of the stub and load in the middle and on the edge.

**Fig 2:**
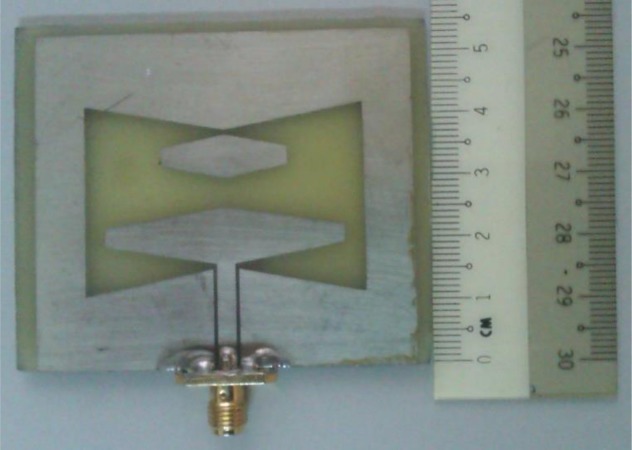
Fabricated Antenna.

Fig 2 presents the fabricated antenna. The proposed antenna has only one side fabrication which makes the antenna easier for fabrication and integration. For the fabrication board we used copper coated FR4 material. The relative permittivity (*ε*_*r*_) of the fiber is 4.3 and it has 1.6mm thickness.

In this paper, the effect of the width, length and indent of the slot area has been studied and analyzed as well as the width and aperture of the stub and the load. These analyses provides a clear indication of the influences of each parameter on the characteristics and reflection coefficient (*S*_*11*_) or return loss of the antenna. This information can facilitate the final design to comply with our requirements and in tuning the antenna for other purposes.

## Simulation and Measurement Results

**Fig 3:**
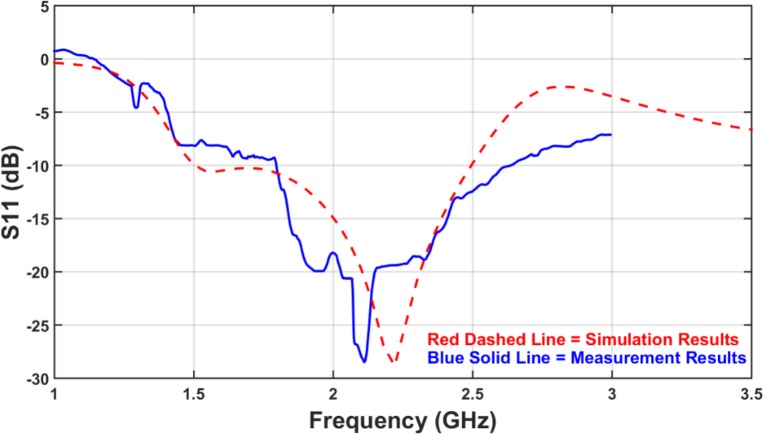
Reflection Coefficient (*S*_*11*_): Simulation versus Measurement.

Fig 3 presents *S*_*11*_ for the designed antenna in both simulation and measurement. It presents the reflection coefficient of the feed port and express the amount of return loss that occurs due to the port mismatch. As long as the *S*_*11*_ is lower than -10dB line, the return loss is acceptable and the antenna is considered to deliver a large enough portion of the power to the environment.

Although the measurement presents lower bandwidth than the simulation results, nevertheless the antenna covers the desired 1.7GHz to 2.6GHz frequency band. The difference between simulation and measurements occurred due to the imperfect situation during the measurement. There are many parameters directly influencing the return loss such as the losses that occur because of the SMA connector and the real amount of the relative permittivity of the substrate. As presented in the figure, if the amount of below -8.5dB are considered to be the desired amount for *S*_*11*_, the achieved bandwidth in simulation and measurement shows no significant difference. Therefore the losses in practice are higher than simulation.

The characteristics of this antenna will be changed by changing the dimensions of the designed geometry. One of the most important characteristics of the antenna is return loss. As shown in the Fig 3, there are two resonant frequencies. If they are near enough to each other and have lower return loss, wide bandwidth property is reachable by the antenna. So the effect of antenna dimensions must be explained for both lower and higher resonant frequencies (*F*_*L*_ and *F*_*H*_ respectively). The characteristics of the design can be tuned by changes applied to the slot as well as the stub and load embedded in the slot geometry.

**Fig 4:**
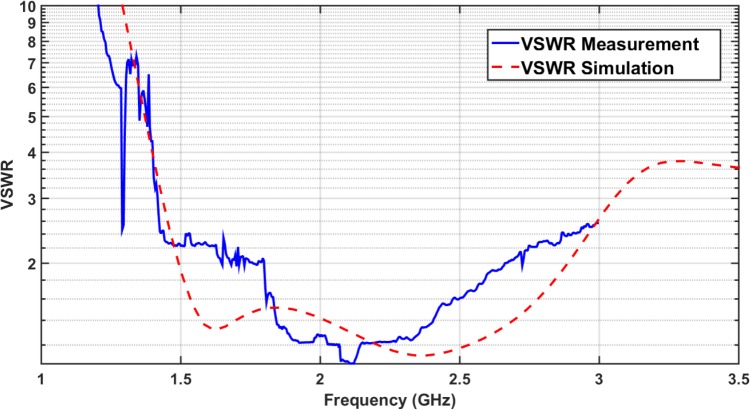
Voltage Standing Wave Ratio (*VSWR*): Simulation versus Measurement.

The reflection coefficient can express voltage standing wave ratio (*VSWR*). The simulation and measurement results for the *VSWR* are demonstrated in Fig 4. It describes the power reflected from the antenna input port.

$$VSWR=\frac{1+|S_{11}|}{1-|S_{11}|}$$

### Effects of Slot Dimensions

**Fig 5:**
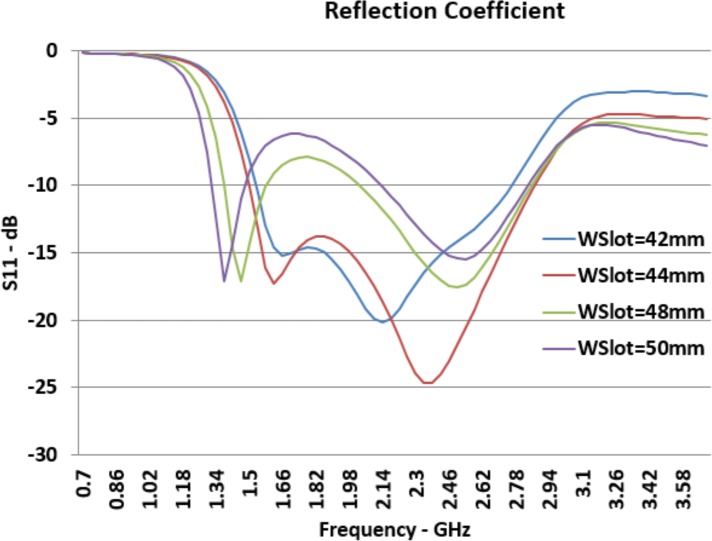
Effect of *W*_*Slot*_ Changes on Reflection Coefficient (*S*_*11*_).

Fig 5 presents the reflection coefficient (*S*_*11*_) of the antenna port for different sizes of *W*_*Slot*_. According to the presented graph, it is immediately obvious when the amount of *W*_*Slot*_ size increases, the *F*_*L*_ decreases a great deal and the F_H_ changes slightly. This potentially indicates that by increasing the width of the slot, the bandwidth will increase.

Another effect is in the middle of *S*_*11*_ graph and it can be seen for a larger width of the slot, the peak between *F*_*L*_ and *F*_*H*_ becomes closer to -10dB and may pass this line and become higher than -10*dB*. Consequently, increment of the slot width in antenna geometry can cause a band-stop between *F*_*L*_ and *F*_*H*_. Effects of changing in slot width on lower resonant frequency are more significant than effects on higher resonant frequency.

**Fig 6:**
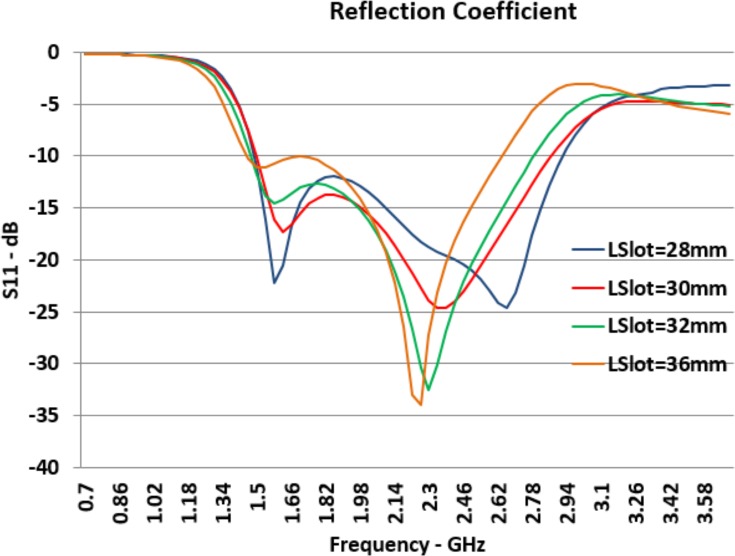
Effect of *L*_*Slot*_ Changes on Reflection Coefficient (*S*_*11*_).

Fig 6 shows the effect of the *L*_*Slot*_ changes on the reflection coefficient. *L*_*Slot*_ is the Bow-Tie slot length and when it increases, both *F*_*L*_ and *F*_*H*_ decrease. The changing of *F*_*H*_ has been significant while changing of *F*_*L*_ has been minimal. Consequently, by increasing the slot length, the bandwidth of the antenna remains almost constant or decreases slightly. By means of the slot length, we can control the working frequency band of the antenna. This can be shifted to the higher or lower bands by decreasing and increasing slot length respectively. The total bandwidth of the antenna will not change significantly.

On the other hand, when slot length increases, return loss at *F*_*L*_ moves closer to the -10*dB* line. The return loss changes at *F*_*H*_ are contradictory. Therefore, increasing of the slot length causes more reflection at the feed port for the lower resonant frequency while it makes lower reflection at higher resonant frequency.

### Effects of the Stub Dimensions

Stub dimension significantly affects the performance and characteristics of the designed antenna. The stub has two apertures (inside and outside) and a width all of which require study.

**Fig 7:**
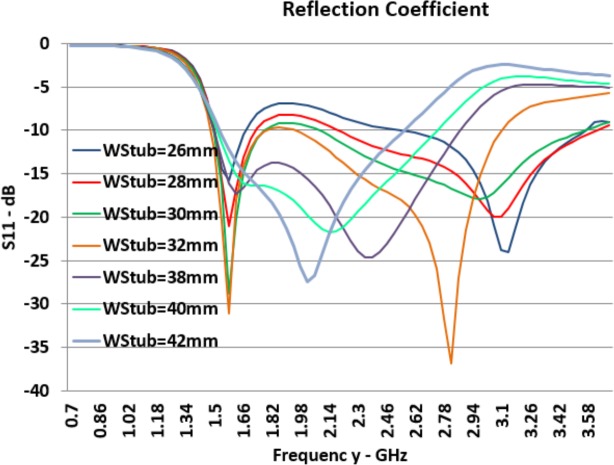
Effect of *W*_*Stub*_ Changes on Reflection Coefficient (*S*_*11*_).

*W*_*Stub*_ is the width of the stub which is located inside the Bow-Tie slot area. Graphs presented in Fig 7 shows the increment of stub width has no significant effect on *F*_*L*_ while it moves *F*_*H*_ to lower frequencies significantly; thus, by increasing the width of the stub, potentially the bandwidth potentially becomes smaller and obviously the center frequency shifts to the lower range.

As it can be seen in Fig 7, by means of increasing the stub width, the reflection coefficient at lower resonant frequency (*F*_*L*_) becomes smaller while it has the inverse effect at the higher resonant frequency.

**Fig 8:**
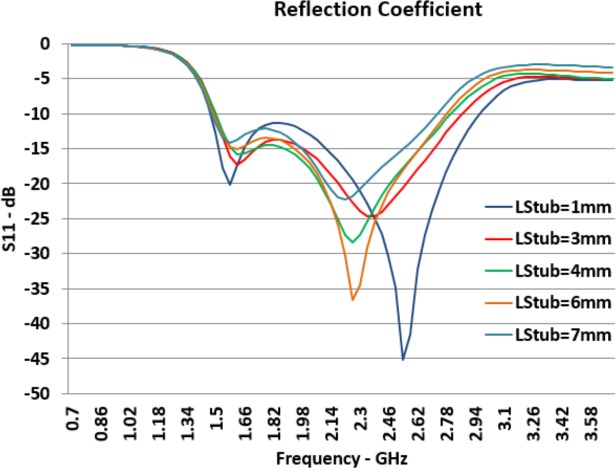
Effect of *L*_*Stub*_ Changes on Reflection Coefficient (*S*_*11*_).

*L*_*Stub*_ presents the size of the outer aperture of the stub. Fig 8 presents the effect of changes in the size of this aperture on the return loss of the designed antenna. When the outer aperture of the stub component increases, *F*_*H*_ became smaller and *F*_*L*_ becomes larger; consequently, the bandwidth has been reduced. Increasing the size of the outer aperture has contradictory effects on the return loss at *F*_*L*_ and *F*_*H*_. Although the increasing of *L*_*Stub*_ has little effect on the *S*_*11*_ at lower resonant frequency and makes the reflection coefficient closer to the -10*dB* line, it has a significant effect in the reverse direction on *S*_*11*_ at the lower resonant frequency.

**Fig 9:**
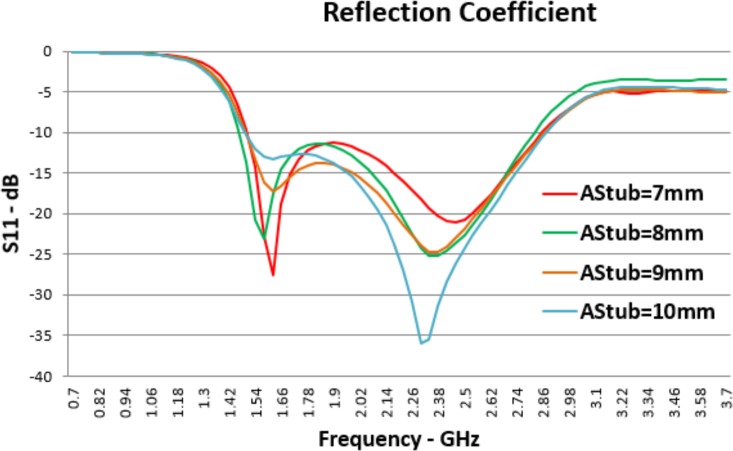
Effects of *A_Stub_* Changes on Reflection Coefficient (*S*_*11*_).

The effect of changing in *A*_*Stub*_ size has been presented in the graphs of Fig 9. It is immediately obvious that these changes do not have a significant effect on higher and lower resonant frequencies but they have effects on return loss at both higher and lower resonance frequencies. Increasing inner aperture size causes lower *S*_*11*_ at higher resonant frequency while it makes a contradictory effect at lower resonant frequency. If the size of *A*_*Stub*_ becomes larger than what is presented, it can provide a band stop in the middle of the antenna bandwidth. So *A*_*Stub*_ can be used to tune the reflection coefficient in both *F*_*L*_ and *F*_*H*_ and also provide a band stop.

### Effects of the Load Element Dimensions

**Fig 10:**
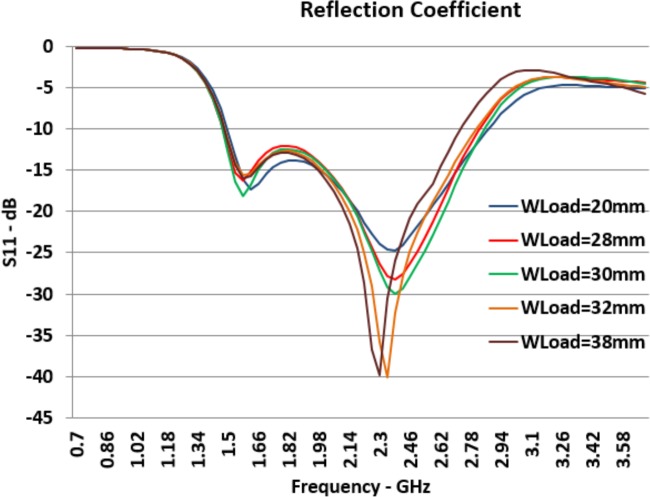
Effects of *W*_*Load*_ Changes on Reflection Coefficient (*S*_*11*_).

*W*_*Load*_ is the width of the load component which is located on top of the stub. *W*_*Load*_ determines the total width of the load and it can be used as a tuning component. By increasing the *W*_*Load*_, *F*_*H*_ reduces slightly and there is no significant change in *F*_*L*_. The return loss at *F*_*H*_ decreases while *W*_*Load*_ became larger. So the width of this tuning load can be used for controlling the reflection coefficient of the higher resonant frequency with no significant change in the bandwidth. This is concluded from the graphs presented in Fig 10.

**Fig 11:**
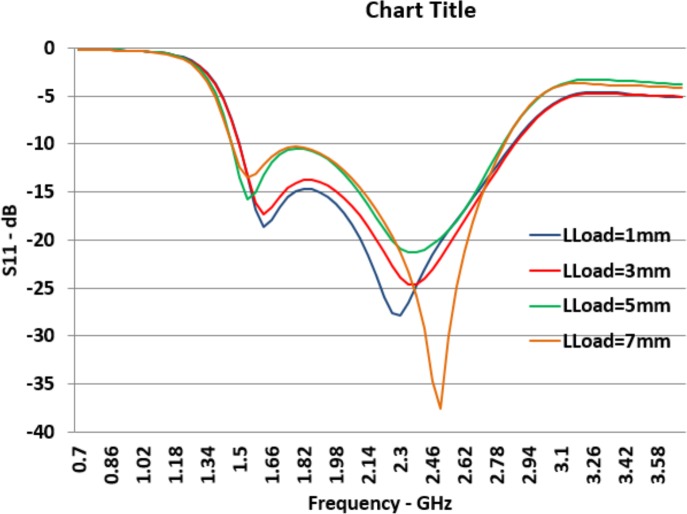
Effects of *L*_*Load*_ Changes on Reflection Coefficient (*S*_*11*_).

The graphs presented in Fig 11 shows the reflection coefficient for different sizes of the *L*_*Load*_. *L*_*Load*_ presents the outer aperture of the load. Increasing the *L*_*Load*_ provides slight changes at both higher and lower resonance frequencies and they are contradictory. If the outer aperture size increases, *F*_*L*_ shifts right slightly and *F*_*H*_ shifts a bit left. Consequently, the working bandwidth of the designed antenna becomes a bit smaller. The reflection coefficient graph at both higher and lower resonant frequencies moves higher. So, this parameter of the antenna can be utilized to create a band stop in the middle of the bandwidth which may be used for some applications to prevent interferences.

**Fig 12:**
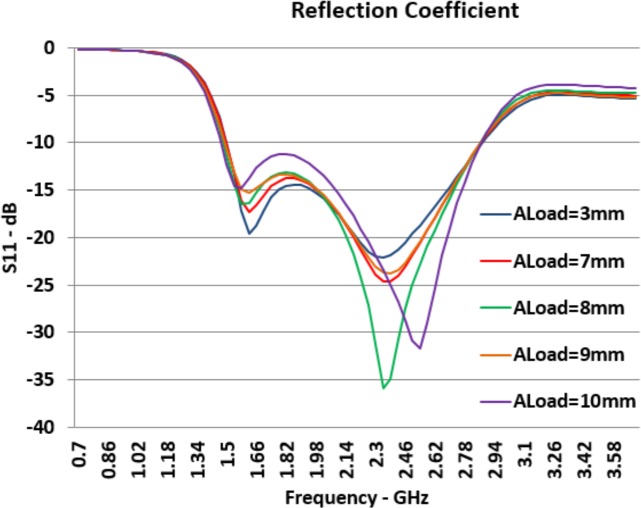
Effects of *A*_*Load*_ Changes on Reflection Coefficient (*S*_*11*_).

*A*_*Load*_ determines the inner aperture of the load component. Graphs presented in Fig 12 show that this aperture can be used to control the return loss of the antenna in its working bandwidth. As observed from graphs, when *A*_*Load*_ increases, *S*_*11*_ at lower resonance frequency moves closer to -10*dB* line and moves higher.

### Radiation Pattern

**Fig 13:**
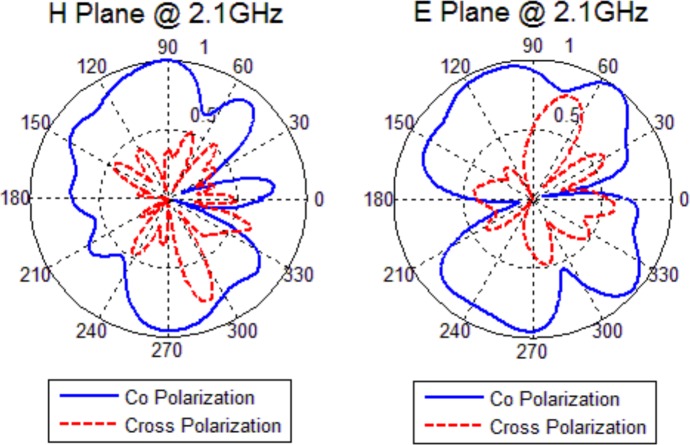
Measured Radiation Patterns at 2.1 *GHz*.

**Fig 14:**
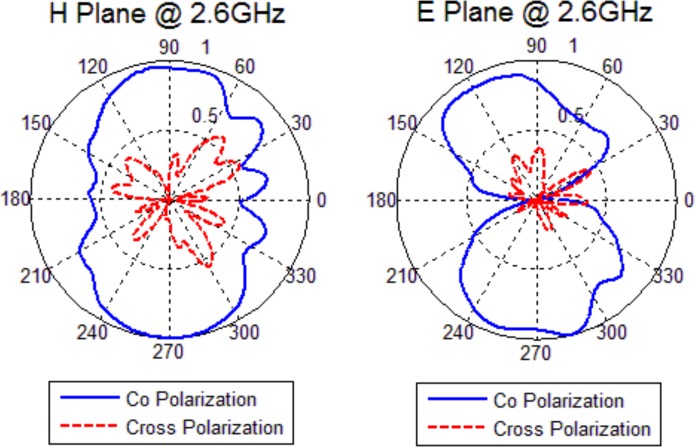
Measured Radiation Patterns at 2.6 *GHz*.

The radiation patterns of the fabricated antenna have been measured in an anechoic chamber in two principal planes: E-Plane and H-Plane. The results are normalized to the maximum value of the resonant frequency and they are shown in Fig 13 and Fig 14. The solid line and the dashed line are co-polarized and cross-polarized components, respectively.

It can be observed that the antenna exhibits approximately omni-directional radiation patterns for H-plane. Because the wavelength is much larger than the antenna size at working frequency, the radiation pattern in E-plane is nearly symmetrical. Although the electrical length increases with higher frequencies, the evident asymmetry in the radiation pattern occurs because of the *CPW* feeding structure. This effect becomes smaller at higher resonant frequency. When the frequency increases, higher order harmonic introduces to patterns and patterns become more directional but still remain almost omni-directional. The measured radiation patterns of the antenna are similar to those of a typical monopole antenna.

## Analysis

The antenna consists of three major components: slot area, stub and load. The dimensions of these components enforce changes in antenna characteristics and its performance effectively. Any optimum design of the antenna needs a correct and in-range initial approximation. According to the parametric studies of the antenna, initial values for the size of each part are proposed as presented in Table 1.

**Table 1:** Initial Design Approximations.

Component		Min Value	Max Value
Slot	Width	λ4	3λ8
	Length	λ8	λ4
Stub	Width	λ8	3λ8
	Outer Aperture	3% of SL*	25% of SL
	Inner Aperture	20% of SL	33% of SL
Load	Width	λ8	λ4
	Outer Aperture	3% of SL	25% of SL
	Inner Aperture	10% of SL	33% of SL

* SL: Total Slot Length.

As it can be seen in the Table 1, slot area size is proportional to the wavelength and its dimension interval limited between two amounts that are a fraction of the wavelength. The width of the slot area of the antenna can be at least a quarter of the wavelength and at most 3λ8. The length of the slot area could be between an octant of the wavelength to a quarter of the wavelength.

As shown, the width of the stub and the load are proportional to the wavelength as well. Nevertheless, aperture size of the stub and the load is a percentage of the slot length. In addition, it is necessary for the apertures not to exceed λ8.

Table 1 can be used for the approximation of the dimensions. These approximations can be used as a starting point for the antenna design to reach the desired frequency band. These approximated dimensions achieve a frequency band which is near to our desired band and they are not the exact size of the antenna. After that, the dimensions must be tuned to get better performance and achieve the exact frequency band for our purposes. Indeed, conditions mentioned in Table 1 are necessary, but are not sufficient. As was elaborated in previous sections, regarding the changes in the dimensions of the slot, stub and load, the final tuning can be accomplished by means of our proposed parametric studies for tuning purposes shown in Table 2.

**Table 2:** Effects of size increment on FL, FH and S11.

Effect of Increasing		*FL*	*S11*@*FL*	*FH*	*S11*@*FH*
Slot	*W*_*Slot*_	**←**	**−**	**−**	**−**
	*L*_*Slot*_	**←**	**↑**	**←**	**↓**
Stub	*W*_*Stub*_	**−**	**↓**	**←**	**↑**
	*L*_*Stub*_	**→**	**↑**	**←**	**↓**
	*A*_*Stub*_	**−**	**↑**	**−**	**↓**
Load	*W*_*Load*_	**−**	**−**	**←**	**↓**
	*L*_*Load*_	**→**	**↑**	**←**	**↑**
	*A*_*Load*_	**−**	**↑**	**−**	**−**

←: Frequency shifts lower.

↑: S11 graph moves higher.

→: Frequency shifts higher.

↓: S11 graph moves lower.

## Conclusion

This paper proposed a *CPW*-Fed printed Bow-Tie slot antenna with loaded stub. Effects of changing in the sizes of slot, stub and load dimensions are considered and then a solution is suggested for design purposes. For any antenna design, the designer needs to know the dimension ranges to consider the limitations of the design. The dimension ranges could be determined by means of some initial values at first and then the final design and final values of the dimensions could be achieved by optimizing those initial values. This paper proposed a reasonable initial approximation for the first design (Table 1). A second table is used for fine-tuning for the final design (Table 2). The designed antenna has wideband characteristics and it achieved more than 50% bandwidth around a centre frequency of 2.1 *GHz*.

*CPW*-Fed structure provides wider bandwidth with lower dispersion in comparison with Probe-Fed antennas. This characteristic is desired for many applications. Unipolar configuration of the single sided *CPW*-Fed antenna makes it easy to be implemented during fabrication and to integrate during utilization. This kind of printed antenna complies with the requirements of small size and light weight, so it is very useful for portable systems. Bow-Tie geometry provides radiation characteristics similar to rectangle antenna, but with smaller size. There are loaded stub which is useful for tuning.

The characteristics of the proposed antenna change by hanging in the dimensions of the designed geometry. The proposed antenna achieved our desired bandwidth. As indicated, it can be tunable with loaded stub that are embedded in the slot geometry. The results are proven through simulation, fabrication and measurement.

## Supporting Information

S1 Table This is the Initial Design Approximations. (DOCX)

S2 Table This is the Effects of size increment on FL, FH and S11. (DOCX)

S1 Text This is the results due to changes of L1. (TXT)

S2 Text This is the results due to changes of L5. (TXT)

S3 Text This is the results due to changes of L7. (TXT)

S4 Text This is the results due to changes of L8. (TXT)

S5 Text This is the results due to changes of L9. (TXT)

S6 Text This is the results due to changes of L10. (TXT)

S7 Text This is the results due to changes of Wslot. (TXT)

S8 Text This is the results due to changes of Wstub. (TXT)

S9 Text This is the results due to changes of Wload. (TXT)

S10 Text This is the S11 at final measurement. (TXT)

S11 Text This is the radiation pattern measurement at final measurement. (TXT)

S12 Text This is the reference file to plot Radiation Pattern. (TXT)

## References

[1] Song H, Brandt-Pearce M. Range of Influence and Impact of Physical Impairments in Long-Haul DWDM Systems. J Lightwave Technol. 2013;31(6):846–54.

[2] Song H, Brandt-Pearce M, editors. Range of Influence of Physical Impairments in Wavelength-Division Multiplexed Systems. Global Telecommunications Conference (GLOBECOM 2011), 2011 IEEE; 2011 5–9 Dec. 2011.

[3] Sheng G, Wang Y, Lv Z, Song H. Multiple-antenna systems and multiuser communications: Fundamentals and an overview of software-based modeling techniques. Computers & Electrical Engineering.

[4] Moosazadeh M, Kharkovsky S. Compact and Small Planar Monopole Antenna With Symmetrical L- and U-Shaped Slots for WLAN/WiMAX Applications. Antennas and Wireless Propagation Letters, IEEE. 2014;13:388–91.

[5] Bing G, Xue Shi R, Ying Yin Z, Lin Hua S, Qiu Rong Z. Compact slot antenna for ultra-wide band applications. Microwaves, Antennas & Propagation, IET. 2014;8(3):200–5.

[6] Xia W, Yang Y, Wenquan C. A Novel Dual-Band Printed Monopole Antenna Based on Planar Inverted-Cone Antenna (PICA). Antennas and Wireless Propagation Letters, IEEE. 2014;13:217–20.

[7] Luo LX Y.-L., Xin Z.-Y., and He S.. A Compact CPW-Fed UWB Antenna with GSM, GPS, Bluetooth And Dual Notch Bands Applications. Progress In Electromagnetics Research C. 2013;35:205–19.

[8] Sayidmarie KH, Fadhel YA. A Planar Self-Complementary BOW-Tie Antenna for UWB Applications. Progress In Electromagnetics Research C. 2013;35:253–67.

[9] Shakib MN, Moghavvemi M, Binti Wan Mahadi WNL. A Low Profile Patch Antenna for Ultrawideband Application. Antennas and Wireless Propagation Letters, IEEE. 2015;PP(99):1–.

[10] Hsuan-Yu C, Sim CYD, Ching-Her L. Dual-Band Meander Monopole Antenna for WLAN Operation in Laptop Computer. Antennas and Wireless Propagation Letters, IEEE. 2013;12:694–7.

[11] Rathore A, Nilavalan R, AbuTarboush HF, Peter T, editors. Compact dual-band (2.4/5.2GHz) monopole antenna for WLAN applications. Antenna Technology (iWAT), 2010 International Workshop on; 2010 1–3 March 2010.

[12] Panda JR, Kshetrimayum RS, editors. A printed inverted double L-shaped dual-band monopole antenna for RFID applications. Applied Electromagnetics Conference (AEMC), 2009; 2009 14–16 Dec. 2009.

[13] Horng-Dean C, Jin-Sen C, Yuan-Tung C. Modified inverted-L monopole antenna for 2.4/5 GHz dual-band operations. Electronics Letters. 2003;39(22):1567–8.

[14] Liu WC, Chen JK. Dual-band twin stepped-patch monopole antenna for WLAN application. Electronics Letters. 2009;45(18):929–31.

[15] Yen-Liang K, Kin-Lu W. Printed double-T monopole antenna for 2.4/5.2 GHz dual-band WLAN operations. Antennas and Propagation, IEEE Transactions on. 2003;51(9):2187–92.

[16] Shakib MN, Moghavvemi M, Mahadi WNL. Design of a Compact Planar Antenna for Ultra-wideband Operation ACES JOURNAL. 2015;30(2):222–9.

[17] Yang J, Wang H, Lv Z, Wang H. Design of Miniaturized Dual-Band Microstrip Antenna for WLAN Application. Sensors (Basel, Switzerland). 2016;16(7):983. 10.3390/s16070983 PMC4970034 27355954

[18] Kavya A, Poornima V, Alex ZC, Shambavi K, editors. Design of a miniaturized dual band patch antenna for WLAN applications. Electronics and Communication Systems (ICECS), 2015 2nd International Conference on; 2015 26–27 Feb. 2015.

[19] Mandal T, Das S. An optimal design of CPW-fed UWB aperture antennas with wimax/WLAN notched band characteristics. Progress In Electromagnetics Research C. 2013;35:161–75.

[20] Gao G-P, Mei Z-L, Li B-N. Novel circular slot UWB antenna with dual band-notched characteristic. Progress In Electromagnetics Research C. 2010;15:49–63.

[21] Yi-Cheng L, Kuan-Jung H. Compact Ultrawideband Rectangular Aperture Antenna and Band-Notched Designs. Antennas and Propagation, IEEE Transactions on. 2006;54(11):3075–81.

[22] Sorbello G, Pavone M, Russello L. Numerical and experimental study of a rectangular slot antenna for UWB communications. Microwave and Optical Technology Letters. 2005;46(4):315–9.

[23] Shafiei MM, Moghavvemi M, Mahadi DWNL. Antenna Tackles Wi-Fi and WiMAX. Microwaves and RF. 2015;Jan 16.

[24] Azim R, Islam M, Misran N. Printed circular disc compact planar antenna for UWB applications. Telecommunication Systems. 2013;52(2):1171–7.

[25] Ovi AAN, Chowdhury MMR, Zuboraj RA, Matin MA. Design of Miniaturized Dual Band Microstrip Antenna Loaded With Asymmetric J Slot. Electrical Engineering and Electronic Technology. 2013;2013.

[26] Lestari AA, Yarovoy AG, Ligthart LP. RC-loaded bow-tie antenna for improved pulse radiation. Antennas and Propagation, IEEE Transactions on. 2004;52(10):2555–63.

[27] Waldschmidt C, Palmer KD. Loaded wedge bow-tie antenna using linear profile. Electronics Letters. 2001;37(4):208–9.

[28] Shih-Yuan C, Powen H, editors. A modified bow-tie slot antenna fed by a coplanar waveguide. Antennas and Propagation Society International Symposium, 2004 IEEE; 2004 20–25 June 2004.

[29] Eldek AA, Elsherbeni AZ, Smith CE. Characteristics of bow-tie slot antenna with tapered tuning stubs for wideband operation. Progress In Electromagnetics Research, PIER. 2004;49:53–69.

[30] Yu-De L, Syh-Nan T. Analysis and design of broadside-coupled striplines-fed bow-tie antennas. Antennas and Propagation, IEEE Transactions on. 1998;46(3):459–60.

[31] Kaswiati WS, Suryana J, editors. Design and realization of planar bow-tie dipole array antenna with dual-polarization at 2.4 GHz frequency for Wi-Fi access point application. Telecommunication Systems, Services, and Applications (TSSA), 2012 7th International Conference on; 2012 30–31 Oct. 2012.

[32] Uduwawala D, Norgren M, Fuks P, Gunawardena AW. A deep parametric study of resistor-loaded bow-tie antennas for ground-penetrating radar applications using FDTD. Geoscience and Remote Sensing, IEEE Transactions on. 2004;42(4):732–42.

[33] Birch M, Palmer KD. Optimized bow-tie antenna for pulsed low-frequency ground-penetrating radar. 2002:573–8.

[34] Nishioka Y, Maeshima O, Uno T, Adachi S. FDTD analysis of resistor-loaded bow-tie antennas covered with ferrite-coated conducting cavity for subsurface radar. Antennas and Propagation, IEEE Transactions on. 1999;47(6):970–7.

[35] Shlager KL, Smith GS, Maloney JG. Optimization of bow-tie antennas for pulse radiation. Antennas and Propagation, IEEE Transactions on. 1994;42(7):975–82.

[36] Hadarig RC, de Cos ME, x, lvarez Y, Las-Heras F. Novel bow-tie antenna on artificial magnetic conductor for 5.8 GHz radio frequency identification tags usable with metallic objects. Microwaves, Antennas & Propagation, IET. 2011;5(9):1097–102.

[37] Berge LA, Reich MT, Braaten BD. A Compact Dual-Band Bow-Tie Slot Antenna for 900-MHz and 2400-MHz ISM Bands. Antennas and Wireless Propagation Letters, IEEE. 2011;10:1385–8.

[38] You-Chieh C, Shih-Yuan C, Powen H. A Compact Triband Bow-Tie Slot Antenna Fed by a Coplanar Waveguide. Antennas and Wireless Propagation Letters, IEEE. 2010;9:1205–8.

[39] Shafiei MM, Roslee M, editors. A Compact Slotted Bowtie Patch Antenna. The 2009 International Symposium on Antennas and Propagation (ISAP 2009); 2009 October 20–23; Bangkok, Thailand.

[40] Chen Y-L, Ruan C-L, Peng L. A novel ultra-wideband bow-tie slot antenna in wireless communication systems. Progress In Electromagnetics Research Letters. 2008;1:101–8.

[41] Smith SL, Merkle T, Smart KW, Hay SG, Mei S, Ceccato F. Design Aspects of an Antenna-MMIC Interface Using a Stacked Patch at 71–86 GHz. Antennas and Propagation, IEEE Transactions on. 2013;61(4):1591–8.

[42] Carroll JM, Tilley KA, Kanamaluru S, Chang K. Slot coupling of coplanar waveguide to patch antennas suitable for MMIC applications. Electronics Letters. 1994;30(15):1195–6.

[43] Simons R. Coplanar waveguide circuits, components, and systems. New York: John Wiley; 2001 xx, 439 p. p.

