# Supplementary material for: The Parametric Study and Fine-Tuning of Bow-Tie Slot Antenna with Loaded Stub
Source: PLoS One. 2017 Jan 23;12(1):e0169033. doi: 10.1371/journal.pone.0169033 (PMC5256992; doi:10.1371/journal.pone.0169033)
Supplement: S1 Table — (DOCX) [file pone.0169033.s001.docx]

| Component | | Min Value | Max Value |
| --- | --- | --- | --- |
| Slot | Width | $\frac{\lambda}{4}$ | $\frac{3\lambda}{8}$ |
|  | Length | $\frac{\lambda}{8}$ | $\frac{\lambda}{4}$ |
| Stub | Width | $\frac{\lambda}{8}$ | $\frac{3\lambda}{8}$ |
|  | Outer Aperture | 3% of SL^*^ | 25% of SL |
|  | Inner Aperture | 20% of SL | 33% of SL |
| Load | Width | $\frac{\lambda}{8}$ | $\frac{\lambda}{4}$ |
|  | Outer Aperture | 3% of SL | 25% of SL |
|  | Inner Aperture | 10% of SL | 33% of SL |
