# Supplementary material for: The Parametric Study and Fine-Tuning of Bow-Tie Slot Antenna with Loaded Stub
Source: PLoS One. 2017 Jan 23;12(1):e0169033. doi: 10.1371/journal.pone.0169033 (PMC5256992; doi:10.1371/journal.pone.0169033)
Supplement: S2 Table — (DOCX) [file pone.0169033.s002.docx]

| **Effect of Increasing** | | ***FL*** | ***S11*@*FL*** | ***FH*** | ***S11*@*FH*** |
| --- | --- | --- | --- | --- | --- |
| Slot | *W_Slot_* | **←** | **−** | **−** | **−** |
|  | *L_Slot_* | **←** | **↑** | **←** | **↓** |
| Stub | *W_Stub_* | **−** | **↓** | **←** | **↑** |
|  | *L_Stub_* | **→** | **↑** | **←** | **↓** |
|  | *A_Stub_* | **−** | **↑** | **−** | **↓** |
| Load | *W_Load_* | **−** | **−** | **←** | **↓** |
|  | *L_Load_* | **→** | **↑** | **←** | **↑** |
|  | *A_Load_* | **−** | **↑** | **−** | **−** |
